# Tumor Immunology, toward a Success Story?

**DOI:** 10.3389/fimmu.2015.00065

**Published:** 2015-02-18

**Authors:** Catherine Sautès-Fridman

**Affiliations:** ^1^UMRS 1138 Centre de Recherche des Cordeliers, Département Cancer, Immunologie et Immunopathologie, Paris, France

**Keywords:** immunotherapy, tumor microenvironment, immune escape, immune checkpoints, microbiota

## Proof of Immunosurveillance and the Immunoediting Concept

The beginning of the second millennium marked a turning point for tumor immunity, on the basis of a series of data obtained in mice and in humans. The enhanced susceptibility of immunodeficient mice to the formation of chemically induced or spontaneous tumors clearly showed that the immune system can eliminate nascent tumors (1). A few years later, the investigations showing that high densities of T cells, particularly with a Th1 and cytotoxic polarization, in different locations of a primary tumor, correlate with favorable prognosis for disease-free and overall survival in large series of cancer patients also strongly supported the fact that a natural immune reaction controls invasion and metastasis (2). Most importantly, these results also proved the establishment of a long-term memory against cancers and suggested that it could be reactivated with tumor vaccines. Such breakthroughs had major consequences for the tumor immunity field. They paved the way for a new era for tumor immunology, building on solid foundations, and leaving behind more than 50 years of errances since the concept of tumor immunosurveillance had been put forward. However, tumors develop, invade tissues locally, metastasize in distant organs, and ultimately cancer can be life threatening. During these last 10 years, we also learnt that the immune system “sculpts” tumors, leading to the formation of tumor variants that escape immunosurveillance, and/or inducing suppressive conditions that facilitate tumor outgrowth. Thus, cancer immunotherapy should aim at boosting anti-tumor immunity by breaking immune tolerance or using adoptively transferred expanded tumor infiltrating lymphocytes (already present in the tumor). This last approach has yielded promising results in terminally ill cancer patients.

## Deeper in the Tumor Microenvironment

In parallel and complementary to these studies, it became evident that a tumor is not only an aggregate of cancer cells but also a complex organization that includes blood and lymphatic vessels, stromal cells, and cells of the immune system. Blood vessels carry fluid, oxygen, nutriments, and cells to tumors, and also drive tumor growth through their interaction with the extracellular matrix, and facilitate the formation of niches of tumor stem cells. An accumulating series of evidence suggest that high endothelial venules (HEV), mostly localized at the invasive margin of tumors, allow the entry of lymphocytes into tumors. The latter point is reinforced by the fact that HEV are the only blood vessels associated with a favorable clinical outcome of cancer patients. Lymphocytes can be primed by mature dendritic cells (DC) locally, and then proliferate and differentiate, forming peritumoral lymphoid aggregates. These so-called tertiary lymphoid structures (TLS) (3) share many features with secondary lymphoid organs. Such a cellular organization most likely facilitates the generation and maintenance of local anti-tumor immune responses, protected from the suppressive influences of the tumor milieu. The lymphatic vessels maintain fluid balance and transport soluble and/or cell bound antigens. They are known to play a detrimental role in cancer since they participate to the transport of metastazing tumor cells. However, lymphatic vessels may also contribute to cancer defenses since they allow the exit of activated DC and memory cells (generated in TLS) from the tumor. Their potential diversity still has to be explored.

The distribution and functions of immune cell within tumors have been the subject of intense studies. The immune landscape contains distinct infiltrating immune cell types with distinct densities, prognostic, and predictive significance in tumors (4). Whereas mature DC, M1 macrophages, Th1 CD4^+^ T cells, and cytotoxic CD8^+^ T cells tend to reduce cancer growth in the vast majority of cancers, M2 macrophages, myeloid-derived suppressor cells (MDSC), and neutrophils exert an opposite effect. The Th2 CD4^+^ T cells, Th17 CD4^+^ T cells, Foxp3^+^CD4^+^ regulatory T (Treg) cells can play a pro or anti-tumoral role, depending on the tumor microenvironment. Moreover, beyond its composition, the intra- and peritumoral distribution, the architecture and the functional organization of the immune infiltrate – the immune contexture – play an important role on its capacity to control tumor growth.

## Anti-Checkpoint Immunotherapies, Impressive Responses, and New Combinations

A large body of evidence came from mouse and human studies showing that T cells express multiple inhibitory molecules that dampen their effector functions upon recognition of their ligands. Antibodies that block these interactions were administrated to cancer patients. Several recent studies based on randomized protocols demonstrate their impressive clinical activity in subsets of patients in several cancer types, with little side effects for some of these agents, further illustrating the power of the immune defenses against cancer (5).

## Big Data, New Avenues

In parallel to these seminal studies, the last 10 years have witnessed the development of collections of large public cancer data sets, including those of The Cancer Genome Atlas (TCGA), which provides a comprehensive collection of aberrations in the DNA, chromatin, and RNA of the genomes of thousands of tumors relative to matched normal cellular genomes in 20 different types of human cancers. This unprecedented effort together with the development of whole genome sequencing set the stage for important new data and new analyses regarding many aspects of tumor biology. The characterization of the molecular heterogeneity of tumors leading to the identification of subgroups of patients with distinct survival rates and responses to treatments – including immunotherapies in the near future – set the premises of personalized medicine. Most importantly, the exome sequencing strategies combined to a set of different approaches including T cell epitope prediction now allow the identification, in a relatively short period of time, of the mutated tumor antigens that are recognized by the patient’s immune system (5). Hence, for the first time it is possible to exploit these strategies for an «à la carte»vaccination of patients.

## What Will Come Next?

Commensal microorganisms colonize barrier surfaces. In addition to their role in nutrient degradation, they modulate innate and adaptive immunity. Their regulatory role on innate and adaptive immune responses to tumors, and its consequences on cancer progression and treatment are beginning to be deciphered. Silent retroviruses hosted in lymphocytes can be reactivated and help them to launch a rapid response against pathogens through NFκB activation. Their role in shaping immune responses including those against cancer may be uncovered in the coming years.

Indeed, metabolic pathways regulate immune cell functions. Epidemiological studies have shown links between patient’s metabolism, diet consumption, and cancer incidence. Moreover, there is growing evidence that the dietary sensitive epigenetic changes such as DNA methylation and histone modifications, microRNAs expression can influence cancer development and T cell function. How all these parameters influence the tumor microenvironment and the complex cross-talk between tumor immunity and tumor metabolism are questions that may be addressed in the near future.

Dramatic progresses have been made in the field of tumor immunology during the preceding 15 years. Such advances are being successfully translated to clinics. However, in most cases, only subgroups of patients respond to immunotherapies. Microenvironment-based biomarkers have been identified but their selection and transfer to the bed side are still needed. There is now no doubt that the immune system can be a powerful ally against cancer. The developments in immune intervention strategies are growing rapidly and further progresses are not far away, that will be crucial in cancer treatment for the benefits of the patients.

## Conflict of Interest Statement

The author declares that the research was conducted in the absence of any commercial or financial relationships that could be construed as a potential conflict of interest.
